# Acute blood pressure levels and long‐term outcome in ischemic stroke

**DOI:** 10.1002/brb3.992

**Published:** 2018-05-18

**Authors:** Johan‐Emil Bager, Clara Hjalmarsson, Karin Manhem, Bjorn Andersson

**Affiliations:** ^1^ Department of Internal Medicine Sahlgrenska University Hospital Göteborg Sweden; ^2^ Department of Cardiology Sahlgrenska University Hospital Göteborg Sweden; ^3^ Institute of Medicine Department of Molecular and Clinical Medicine Sahlgrenska University Hospital Sahlgrenska Academy University of Gothenburg Göteborg Sweden; ^4^ The Stroke Unit Department of Internal Medicine Sahlgrenska University Hospital Göteborg Sweden

**Keywords:** blood pressure, brain ischemia, mortality, recovery of function, regression analysis, stroke

## Abstract

**Objectives:**

Elevated blood pressure (BP) is common in acute ischemic stroke, but its effect on outcome is not fully understood. We aimed to investigate the association of baseline BP and BP change within the first day after stroke with stroke severity, functional outcome, and mortality.

**Methods:**

Patients admitted to hospital with acute ischemic stroke (IS) from 15 February 2005 through 31 May 2009 were consecutively included. Acute stroke severity and functional outcome at three and twelve months were investigated using multivariate regression analysis; the association between BP and all‐cause mortality at one, three, and twelve was investigated by Cox proportional hazard regression and Kaplan–Meier survival curves.

**Results:**

A total of 799 patients (mean age 78.4 ± 8.0, 48% men) were included. Higher decreases in systolic and mean arterial blood pressure (ΔSBP and ΔMAP) were associated with decreased 1‐month mortality (ΔSBP: hazard ratio, HR: 0.981; 95% CI: 0.968 – 0.994; *p* = .005), 3‐month mortality (ΔSBP: HR 0.989; 95% CI 0.981 – 0.998; *p*‐value .014), and twelve‐month mortality (ΔSBP: HR 0.989; 95% CI 0.982 – 0.996; *p*‐value .003). Stroke severity was associated with ΔMAP (B coefficient −.46, *p*‐value .011). Higher SBP and MAP on admission were associated with better functional outcome at three (SBP: OR 0.987; 95% CI 0.978 – 0.997; *p*‐value .008 ‐ MAP: OR 0.985; 95% CI 0.971 – 1; *p*‐value .046) and twelve (SBP: OR 0.988; 95% CI 0.979 – 0.998; *p*‐value .015 – MAP: OR 0.983; 95% CI 0.968 – 0.997; *p*‐value .02) months.

**Conclusion:**

In this elderly population, higher BP on arrival to the emergency room (ER) and decrease in BP after the patients’ arrival to the ward were associated with improved functional outcome and reduced mortality, respectively. These results may reflect a regulatory situation in which elevated initial blood pressure indicates adequate response to cerebral tissue ischemia while subsequent blood pressure decrease instead may be a consequence of partial, successful reperfusion.

## 1 INTRODUCTION

Elevated blood pressure (BP) is a common observation in the acute setting in patients with acute ischemic stroke (IS) (Wallace & Levy, 1981). In a study of over 500,000 patients with stroke by Qureshi et al. (2007), 77% of the 276,734 patients with verified ischemic strokes displayed systolic blood pressures (SBP) >140 mmHg at the emergency department. Typically, BP then decreases spontaneously, starting within hours of stroke onset (Britton, Carlsson, & de Faire, 1986; Broderick et al., 1993; Jauch et al., 2013; Wallace & Levy, 1981). It has been hypothesized that elevated BP in stroke patients can be either beneficial or detrimental, by mechanisms of improved cerebral perfusion in ischemic tissue or by edema and complicating hemorrhage, respectively (Britton et al., 1986; Jauch et al., 2013). A study of leptomeningeal collateral circulation in patients with acute IS who received intravenous thrombolysis showed that collateral circulation was better when BP was elevated (Rusanen, Saarinen, & Sillanpaa, 2015).

Kvistad et al. (2013) have reported an inverse association between elevated BP on admission and stroke severity. There was no significant difference, however, in short‐term functional outcome. In a study of 831 patients with IS or transient ischemic attack (TIA) by Carlberg, Asplund, & Hagg (1993) there was no significant association between mean arterial pressure (MAP) on admission and mortality 30 days after admission.

Other studies have linked elevated BP in the acute setting to poor outcome in terms of dependency at 21 days and 6 months, respectively, or early recurrence of stroke and death (Ahmed & Wahlgren, 2001; Leonardi‐Bee, Bath, Phillips, & Sandercock, 2002). A U‐shaped relationship between admission BP and early and late death has been shown, suggesting that both high and low BPs are detrimental (Leonardi‐Bee et al., 2002; Vemmos et al., 2004). Wohlfahrt et al. (2015) too, noted an association between lower BP and death . Recently, a meta‐analysis of several relatively small studies (Kakaletsis et al., 2015) investigated the association between outcome in acute IS and BP measured by ambulatory monitoring methods during the first 24 hr of admission and found that elevated pressures predicted poor outcome, in the form of either death or poor functional outcome.

The importance of change from the baseline BP at admission has also been studied, and correlation between early, spontaneous BP decrease, and lesser stroke severity as well as improved functional outcome at 10 days and 3 months has been suggested (Abboud, Labreuche, Plouin, & Amarenco, 2006; Christensen, Meden, Overgaard, & Boysen, 2002). Furthermore, it has been suggested that positive outcome is associated with SBT decrease when the baseline SBP is in the 110‐170 mmHg range and that, conversely, increase in SBP from baseline generally predicted worse outcome (Ntaios, Lambrou, & Michel, 2011). Blood pressure variability, too, has been associated with early neurological deterioration (Chung et al., 2015).

On the other hand, there are also reports of worse stroke outcome and increased mortality after 3 months in patients with SBP decrease higher than 20 mmHg (Castillo et al., 2004). Furthermore, in the SCAST study, BP decreases through antihypertensive treatment neither reduced mortality, nor improved functional outcome (Sandset et al., 2011). A large Chinese trial (CATIS) was, like the SCAST study, unable to link BP reduction to decreased short‐term mortality or improved functional outcome (He et al., 2014). Interestingly, the 3‐month follow‐up of CATIS showed a lower risk of recurrent stroke in patients who received antihypertensive treatment 24‐48 hr after suffering an acute IS (Xu et al., 2017).

A recent meta‐analysis of BP lowering randomized controlled trials, additionally, was unable to clarify whether acute BP decrease is beneficial or not (Liu, Li, Li, Xiong, & Zhao, 2015).

The true significance of BP in the acute setting of stroke still proves elusive and the purpose of this study, therefore, was to further investigate the association of baseline BP and BP changes with short‐ and long‐term mortality, acute stroke severity, and long‐term functional outcome. To our knowledge, no other studies encompassing over 700 patients have described follow‐up data at twelve months.

## 2 MATERIALS AND METHODS

### 2.1 Study design and population

This study is a prospective analysis of consecutively included patients with acute IS admitted to the Stroke Ward of the medical department of Sahlgrenska University Hospital from 15 February 2005 through 31 May 2009. The endpoints of interest were as follows: acute stroke severity, functional outcome, and survival. Because of the organizational structure at Sahlgrenska University Hospital at the time of the study, younger stroke patients (<65 years) generally received treatment at the Stroke Ward of the neurological department, rather than that of the medical department. The neurological department also treated patients who underwent thrombectomy or received thrombolytic therapy. Thus, younger stroke patients and patients who received more invasive therapy were not included in our material. Patient demographics and clinical history were collected consecutively and recorded in a database. The study was approved by the Regional Ethical Review Board in Gothenburg.

### 2.2 Data collection

Standardized forms were used to obtain relevant medical history data from patients via interview or collected from medical records. When a patient was unable to answer for him or herself, a knowledgeable proxy was interviewed instead.

Preexisting conditions, for example, hypertension, were considered present if patients, proxies, or medical records reported them as such or if the patients were treated with drugs targeting the condition (e.g., antihypertensive drugs). The same principle was applied for diabetes mellitus, atrial fibrillation, previous stroke, myocardial infarction, and congestive heart failure.

Standard techniques were utilized for laboratory analyses. Computed tomography scans were performed on admission. Stroke diagnosis was assessed in accordance with the World Health Organization's definition (1988).

The severity of the stroke was defined at the ward using the National Institutes of Health Stroke Scale (NIHSS) by specially trained personnel (Brott et al., 1989; Konig et al., 2008). Stroke subtypes were classified according to the Trial of Org 10172 in Acute Stroke Treatment (TOAST) (Adams et al., 1993). Functional outcome was measured after three and twelve months with modified Rankin Scale (mRS), either in conjunction with follow‐up visits, via telephone, or through assessment of medical records (Bonita & Beaglehole, 1988). Mortality data were obtained electronically from the Swedish Population Register, in which all deaths are registered.

Blood pressure values were measured manually using a sphygmomanometer and stethoscope, where the appearance and disappearance of Korotkoff sounds represented systolic and diastolic pressures, respectively. The measurements were made once per measuring occasion by regular, trained hospital personnel, predominantly nurses or assistant nurses. BP was first recorded on arrival to the emergency room (ER), and subsequently on arrival to the ward, the latter measurement occurring no later than 12 hr after admission to the ER. Systolic and diastolic BPs were measured in the right arm, in a supine position. If, however, the right arm was afflicted by palsy, pressures were measured in the left arm. BPs were measured on admittance to the ward and on days 1, 2, 3, 7, and at discharge. At the time of the study, local guidelines mandated acute lowering of blood pressure with antihypertensive therapy in patients with IS when BP was ≥220 mmHg.

Blood pressure values are presented in the form of systolic, diastolic, and MAP. The MAP values were obtained from SBP and diastolic BP (DBP) values through the following equation: MAP = (SBP*1/3) + (DBP*2/3). Difference in SBP values (ΔSBP) was defined as the SBP value on arrival to the ward, subtracted from the SBP value on arrival to the ER. The same principle was applied for calculating ΔMAP. DBP was not included independently in our analyses because previous work suggests that SBP is a better predictor of events in elderly patients (Franklin et al., 1997; Mancia et al., 2013; Vishram et al., 2012). In addition, DBP was indirectly included as a component of MAP.

### 2.3 Outcomes

Our primary endpoints were acute stroke severity (measured by NIHSS) and all‐cause mortality during the acute phase, at three, and at twelve months. A secondary endpoint was functional outcome, measured by modified Rankin Scale (mRS), at three and 12 months, respectively.

### 2.4 Statistical analysis

The associations between BP values and mortality and functional outcome were investigated using Cox proportional hazard regression and binary logistic regression, respectively. In both analyses, age, sex, NIHSS score in the acute setting, congestive heart failure, diabetes mellitus (type 1 and type 2), and history of IS were used as covariates. In the analyses involving ΔSBP and ΔMAP, SBP and MAP, respectively, were also included as covariates. For the binary logistic regression, values for mRS were categorized into a dichotomous variable, in which a score of 0‐2 was considered a positive outcome and a score of 3‐5 was considered a negative outcome. Patients with mRS scores of 6, which equals death, were excluded from the analysis.

For the analysis of associations between BP values and NIHSS score in the acute setting, linear regression was used. In this analysis, age, sex, and history of IS were used as covariates.

We also performed a separate regression analyses for positive and negative ΔBP values, respectively, to further clarify the significance of increases or decreases in BP, and the covariates were the same as in the Cox proportional hazard regression mentioned above. All statistical analyses were performed with the software IBM SPSS Statistics, version 24 (IBM Corporation, Armonk, New York, United States). Results were considered statistically significant if *p*‐values <.05.

## 3 RESULTS

### 3.1 Baseline characteristics

In total, 799 patients were included. Demographic data, medical history, baseline BP, and laboratory values are presented in Table 1. TOAST distribution is shown in Table 2.

**Table 1:** Baseline characteristics of the study population

	Ischemic stroke (*n* = 799)
Baseline values	
Age, years	78.4 ± 8.0
Systolic BP on arrival at ER, mmHg	166 ± 29.7
Diastolic BP on arrival at ER, mmHg	92 ± 16.2
Mean arterial pressure on arrival at ER, mmHg	116 ± 18.4
SBP decrease, mmHg	7.4 ± 17.5
MAP decrease, mmHg	6.1 ± 28.5
SBP decrease, %	58
MAP decrease, %	66
NIHSS, points	7.3 ± 6.7
Cholesterol, mmol/L	4.9 ± 1.2
LDL cholesterol, mmol/L	2.9 ± 1.0
HDL cholesterol, mmol/L	1.5 ± 0.5
Triglycerides, mmol/L	1.3 ± 0.6
Time from symptom onset to arrival at ER, hr	2.6 ± 2.9
Time from arrival at ER to admission at stroke ward, hr	5.3 ± 3.4
History	
Male, %	48
Ischemic stroke, %	27
Intracerebral hemorrhage, %	3
Transitory ischemic attack, %	8
Hypertension, %	57
Atrial fibrillation, %	31
Myocardial infarction, %	15
Heart failure, %	12
Diabetes mellitus (type 1 and 2), %	17

BP, blood pressure; ER, emergency room; SBP, systolic blood pressure; MAP, mean arterial pressure; NIHSS, The National Institutes of Health Stroke Scale.

Demographic data. Data are mean ± *SD* or percentages. Percentages reflect valid data entries, which may be lower than the total number of patients in each group.

**Table 2:** Trial of Org 10172 in Acute Stroke Treatment (TOAST) classification

	*n* = 795^a^
Large‐artery atherosclerosis, %	26
Cardioembolism, %	35
Small‐vessel occlusion, %	25
Stroke of other determined etiology, %	1
Stroke of undetermined etiology, %	14

Percentages reflect valid entries. Rounding may cause total percentage to exceed 100%.

a *N* = 795 because TOAST classification was missing in four patients.

### 3.2 Short‐term outcomes

#### 3.2.1 Mortality

All in all, 70 patients had died at 1‐month follow‐up. Both ΔSBP and ΔMAP displayed significant association with short‐term mortality whereby BP decrease on arrival to the ward was associated with lower mortality. Data are displayed in Table 3.

**Table 3:** Multivariate analysis of association between blood pressure levels and mortality at follow‐up

	1‐month follow‐up			3‐month follow‐up			12‐month follow‐up		
	HR	95% CI	*p*	HR	95% CI	*p*	HR	95% CI	*p*
BP at ER									
SBP	1	0.98–1.012	.965	1	0.99–1.009	.923	0.994	0.986–1.002	.154
MAP	1.007	0.988–1.098	.465	1.003	0.987–1.020	.725	0.993	0.980–1.006	.295
BP change									
ΔSBP	0.981	0.968–0.994	.005	0.989	0.981–0.998	.014	0.989	0.982–0.996	.003
ΔMAP	0.961	0.940–0.983	<.001	0.97	0.953–0.987	.001	0.982	0.97–0.993	.002

BP, blood pressure; ER, emergency room; HR, hazard ratio; SBP, systolic blood pressure; MAP, mean arterial pressure.

Age, sex, NIHSS score in the acute setting, congestive heart failure, diabetes mellitus (type 1 and type 2), and history of ischemic stroke (IS)were used as covariates. In the analyses of ΔSBP and ΔMAP, SBP and MAP, respectively, were also included as covariates.

#### 3.2.2 Stroke severity

In linear regression analysis, stroke severity, as measured by NIHSS, was associated with ΔMAP (B coefficient −.05, *p*‐value .01).

### 3.3 Long‐term outcome – Three and twelve months

#### 3.3.1 Mortality

**Figure 1:**
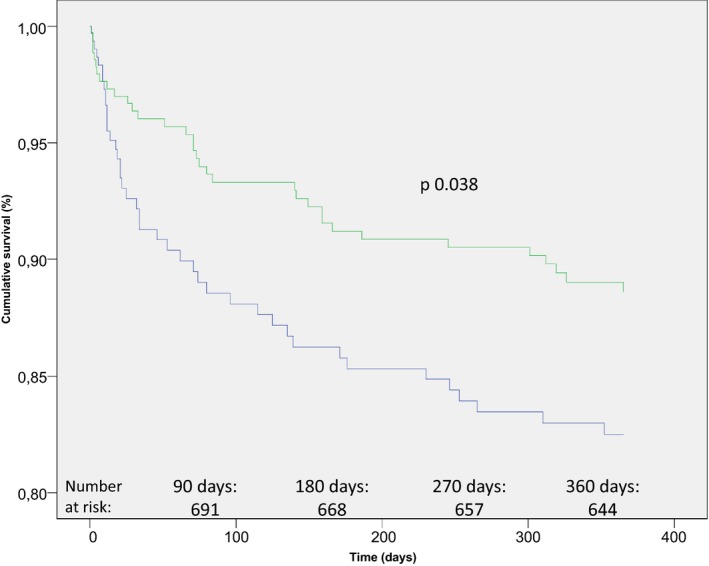
Kaplan–Meier plot displaying percentage of survivors after twelve months, stratified by increase or decrease in systolic blood pressure (SBP). The blue graph represents patients with an increase in systolic blood pressure (SBP) on arrival to the stroke ward, when compared to the admission blood pressure at the emergency room. The green graph represents patients with a corresponding decrease in SBP. Age, sex, National Institutes of Health Stroke Scale (NIHSS) score in the acute setting, congestive heart failure, diabetes mellitus (type 1 and type 2), SBP, and history of ischemic stroke (IS) were used as covariates

In total, 113 (14%) and 157 (20%) patients had died at 3‐month and twelve‐month follow‐up, respectively. We found a significant association between ΔBP and mortality at both three and twelve months. Decrease in BP, both in the form of SBP and in the form of MAP, was thus associated with lower mortality rates. Data are shown in Table 3. Furthermore, in the dichotomized analysis based on MAP increase/decrease, we found increase in BP to be related to significantly higher mortality risk at twelve months, see Table 4. Kaplan–Meier survival curves by SBP increase/decrease are shown in Figure 1.

**Table 4:** Multivariate analysis of association between either increase or decrease in blood pressure and mortality at follow‐up

	1‐month follow‐up			3‐month follow‐up			12‐month follow‐up		
	HR	95% CI	*p*	HR	95% CI	*p*	HR	95% CI	*p*
SBP increase	1.005	0.983–1.027	.68	1.012	0.996–1.028	.145	1.005	0.989–1.021	.561
MAP increase	1.02	0.997–1.054	.242	1.022	0.991–1.053	.163	1.027	1.002–1.053	.035
SBP decrease	0.973	0.941–1.005	.098	0.981	0.957–1.006	.142	0.987	0.966–1.009	.248
MAP decrease	0.947	0.900–0.998	.041	0.946	0.909–0.984	.006	0.966	0.935–0.998	.035

HR, hazard ratio; SBP, systolic blood pressure; MAP, mean arterial pressure.

Age, sex, NIHSS score in the acute setting, congestive heart failure, diabetes mellitus (type 1 and type 2), history of ischemic stroke (IS) and SBP or MAP, respectively, were used as covariates.

#### 3.3.2 Functional outcome

There was a significant association between SBP on admission and functional outcome at both three and twelve months, suggesting that higher initial SBP is linked to better long‐term functional outcome. Data are shown in Table 5. MAP, too, was significantly associated with functional outcome at three and twelve months (see Table 5). No significant associations were found between BP change and functional outcome.

**Table 5:** Multivariate analysis of associations between blood pressure levels and functional outcome

	3‐month follow‐up			12‐month follow‐up		
	OR	95% CI	*p*	OR	95% CI	*p*
BP at ER						
SBP	0.987	0.978–0.997	.008	0.988	0.979–0.998	.015
MAP	0.985	0.971–1	.046	0.983	0.968–0.997	.002
BP change						
ΔSBP	1	0.989–1.012	.954	1.007	0.996–1.019	.213
ΔMAP	0.998	0.980–1.017	.842	1.011	0.992–1.031	.259

BP, blood pressure; ER, emergency room; SBP, systolic blood pressure; MAP, mean arterial pressure.

Age, sex, NIHSS score in the acute setting, congestive heart failure, diabetes mellitus (type 1 and type 2) and history of ischemic stroke (IS) were used as covariates. In the analyses of ΔSBP and ΔMAP, SBP and MAP, respectively, were also included as covariates.

## 4 DISCUSSION

Our results thus demonstrate that a decrease in BP, as measured by a drop in either SBP or MAP when comparing BP on arrival to the ER to BP on arrival to the ward, is associated with reduced mortality. The correlation between BP decrease (ΔSBP and ΔMAP) and mortality was significant in both short‐term and long‐term mortality analyses.

We also show that a higher admittance SBP or MAP level, measured on arrival to the ER, is significantly associated with better long‐term functional outcome.

The mean age of our patients was considerably higher than in previous studies, and we therefore believe our data shed some light on the importance of BP in the acute setting in this older and thus previously unstudied cohort.

We found no significant association between BP on arrival and mortality, neither short‐term nor long‐term. Unlike Wohlfahrt et al. (2015), however, we adjusted for congestive heart failure (CHF) in our mortality analyses. In a separate analysis (*t* test data not shown), our heart failure patients had significantly lower blood pressure than patients without CHF. The inclusion of CHF as a covariate in our regression analyses might thus explain why we did not see an association between lower blood pressure and mortality.

Our findings, thus, suggest a protective effect of larger BP decreases as well as higher admission BP, where larger BP decrease is associated with lower mortality and higher admission BP is associated with better functional outcome. These findings are in line with the hypothesis that BP elevation in stroke is a physiological, beneficial response to increased demands of perfusion (Britton et al., 1986). They are also in accordance with results from several other groups, which have demonstrated beneficial associations between early BP decrease and stroke severity as well as functional outcome (Abboud et al., 2006; Christensen et al., 2002; Ntaios et al., 2011). The combination of finding that both larger BP decrease and higher admission BP decrease the risk of negative outcome may seem paradoxical, but may reflect the situation in acute stroke in which demand of perfusion initially is increased but, subsequently, wanes as a result of reperfusion and tapering ischemia. Interestingly, the separate analysis of patients with MAP increases on arrival to the ward shows that this scenario is associated with greater risk of long‐term mortality, as suggested in previous work (Ntaios et al., 2011).

Several studies have explored the effects of antihypertensive therapy in the acute setting of stroke; none, however, has convincingly linked pharmaceutical BP lowering to positive effects on outcome (Kaste et al., 1994; Potter et al., 2009; Sandset et al., 2011). A subgroup analysis of the CATIS trial suggested that initiating antihypertensive therapy 24‐48 hr after IS onset might decrease the rate of stroke recurrence at 3‐month follow‐up (Xu et al., 2015). As the authors point out, however, their results should interpret with some caution, as the treatment group had both a significantly lower blood pressure and significantly higher usage of antihypertensive medication than the control group. All in all, these results, too, fit with the hypothesis that lowering of blood pressure should be avoided in the setting of acute ischemic stroke.

There are some limitations to this study, one being that BP values were measured only once rather than twice within 5 min, which would have been more ideal. Patients who received thrombolytic therapy, furthermore, were not included in the analysis, but at the time of the study, these patients constituted only 6.6% of total stroke patients and an addition of them to the data would thus be unlikely to have more than a marginal effect on the results. Additionally, we did not adjust for whether patients received antihypertensive treatment in the acute setting or not. According to local guidelines at the time of the study, 4% of the patients had a BP of ≥220 mmHg and were thus eligible for acute antihypertensive treatment. Finally, the paucity of younger patients in our material also limits the external validity of our study in that part of the population.

One strength of our study is excellent follow‐up data, in part due to reliable census data for mortality, but also comprising mRS values for 98% and 92% of patients at three and twelve months, respectively. As mentioned, our study also establishes that BP in the acute setting is an important factor in an older patient cohort (mean age 78 years). Another strength is the reasonably large patient sample (*n* = 799) and a systematic, consecutive, and unbiased patient inclusion method.

In conclusion, our study shows that a greater decrease in BP on the patients’ admission to the ward and higher BP on arrival to the emergency room is associated with reduced mortality and improved functional outcome, respectively. Conversely, increases in MAP are a predictor of increased long‐term mortality. These results may reflect a regulatory situation in which elevated initial BP indicates adequate response to cerebral tissue ischemia while greater subsequent BP decrease may be a consequence of relatively successful reperfusion.

## CONFLICT OF INTEREST

The authors declare no conflict of interest.
